# Attitudes towards Use of High-Importance Antimicrobials—A Cross-Sectional Study of Australian Veterinarians

**DOI:** 10.3390/antibiotics11111589

**Published:** 2022-11-10

**Authors:** Anna Sri, Kirsten E. Bailey, James R. Gilkerson, Glenn F. Browning, Laura Y. Hardefeldt

**Affiliations:** 1 Asia-Pacific Centre for Animal Health, Melbourne Veterinary School, Faculty of Veterinary and Agricultural Sciences, University of Melbourne, Parkville, VIC 3010, Australia; 2 National Centre for Antimicrobial Stewardship, Peter Doherty Institute, Parkville, VIC 3052, Australia

**Keywords:** stewardship, antimicrobial resistance, critically important antimicrobials, antibiotic, companion animals, food-producing animals, restrictions, rating system

## Abstract

The timely implementation of antimicrobial stewardship interventions could delay or prevent the development of higher levels of antimicrobial resistance in the future. In food-producing animals in Australia, high-importance antimicrobials, as rated by the Australian Strategic and Technical Advisory Group (ASTAG), include virginiamycin and third-generation cephalosporins (in individual pigs or cattle). The use of high-importance antimicrobials in companion animals is more widespread and less regulated. There is no national antimicrobial use surveillance system for animals in Australia. Consequently, there is a gap in the knowledge about reasonable use across all sectors of veterinary practice. This study explored attitudes towards the use in veterinary medicine of antimicrobials with high importance to human health, and determined levels of agreement about the introduction of restrictions or other conditions on this use. An online survey was distributed via social media and email from June to December 2020 to veterinarians working in Australia. Of the 278 respondents working in clinical practice, 49% had heard of the ASTAG rating system, and 22% used a traffic light system for antimicrobial importance in their practice. Overall, 61% of participants disagreed that veterinarians should be able to prescribe high-importance antimicrobials without restrictions. If there were to be restrictions, there was most agreement amongst all respondents for only restricting high-importance antimicrobials (73%). There is a need for education, guidance, and practical support for veterinarians for prescribing high-importance antimicrobials alongside any restrictions.

## 1. Introduction

Antimicrobial resistance (AMR) is now well-established as a global problem with profound impacts on nearly all living beings and the environments they inhabit [1]. It is a multigenerational challenge, with increasing levels of morbidity and mortality expected into the future for humans and animals [2,3]. A landmark report released in 2016 estimated that up to 10 million human lives could be lost every year due to AMR by 2050 [1], over half the number of deaths attributed to the COVID-19 pandemic (18.2 million) in the one-year period from January 2020 to January 2021 [4]. In the recent 2021 Global Antimicrobial Resistance and Use Surveillance System report, many countries reported high rates of resistance to high-importance antimicrobials [5]. Given the extremely serious future consequences of AMR and the dynamic situation we face today, it is important that all sectors take responsibility for mitigating the further development of AMR. Timely implementation of antimicrobial stewardship (AMS) interventions could delay or prevent the development of increased levels of AMR in animals and people in the future [6,7].

In Australia, the importance of antimicrobials is determined by the Australian Strategic and Technical Advisory Group on AMR (ASTAG) [8]. The use of high-importance antimicrobials in Australian food-producing animals is limited to streptogramins (virginiamycin only) and third-generation cephalosporins (in individual pigs or cattle only) [8]. The use of fluoroquinolones, gentamicin and colistin has never been permitted in food-producing animals in Australia [8,9,10,11]. As a result of numerous factors—including good regulation and residue monitoring, a predominance of extensive, rather than intensive, animal production systems and the widespread use of biosecurity and vaccination—Australia’s levels of AMR, particularly within domestic animal populations, are relatively low [12,13,14,15]. The low levels of fluroquinolone resistance in the Australian human population compared to other countries could also be a result of the well-regulated use of this class of antibiotics in people and domestic animals in Australia since the early 1990s [12,16,17].

As the development of AMR is a time-sensitive problem, it would be wise to act quickly across all sectors, rather than waiting until evidence is clearer but resistance is more widespread [18]. At present, there is sufficient evidence that all antimicrobial use in domestic animals, humans and agriculture drives resistance at some level [19,20,21,22,23,24]. Therefore, improving the quality of prescribing for therapeutic use and reducing the unreasonable use of antimicrobials is of utmost importance. Unfortunately, assessing the quality of prescribing, particularly in a private veterinary setting, can be difficult. Gaps in knowledge about what is considered ‘reasonable use’ exist across all sectors of veterinary practice. Currently, there is no regular, systematic ongoing surveillance of antimicrobial use or resistance in animals in Australia [25]. There is scope to improve the quality of prescribing in food-producing animals in Australia [26], but more publicly available knowledge about volumes of antimicrobial use and the disease presentations for which these antimicrobials are being used is also required. As the companion animal sector, including horses, has not been subject to the same strict regulations that govern veterinarians treating livestock in Australia, the use of high-importance antimicrobials is common [27,28,29,30,31]. There is also potential for more rapid improvement in prescribing and guideline compliance in these areas of practice because much of the current use of high-importance antimicrobials is not guideline compliant and other treatment options are readily available. These sectors have also not traditionally been the focus of significant educational, public or regulatory pressures to reduce unnecessary antimicrobial use [27,28,32,33].

Restricting antimicrobial use has resulted in improvements in prescribing in a human inpatient setting, particularly when combined with other antimicrobial stewardship (AMS) interventions [16,34,35], and this has also been seen in food-producing animals [6,36,37,38,39]. There has been very little research in Australia on the attitudes of veterinarians about the imposition of restrictions on antimicrobial use. Previous work has shown that veterinarians are concerned about AMR and are prepared to change their prescribing practices, but they face a number of barriers to implementing AMS practices [31]. These barriers include a lack of AMS governance structures, as well as other factors associated with the largely private nature of veterinary practice in Australia [31]. The use of restrictions as an AMS intervention has been rated as ‘not helpful’ in improving prescribing by Australian veterinarians, doctors and dentists [40]; however, other research, particularly from European countries, has suggested that restrictions can result in improved prescribing with no or minimal adverse economic impact [41,42,43] and reduced contamination of the environment with bacteria resistant to antimicrobials or antimicrobial resistance genes [7]. The impact of restrictions also needs to be assessed alongside animal welfare outcomes, as there are significant gaps in understanding these interactions [44].

The purpose of this study was to improve the understanding of the attitudes of Australian veterinarians about antimicrobial use, particularly regarding the use in veterinary medicine of antimicrobials with high importance to human health. An improved understanding of these attitudes will enable more targeted and effective AMS interventions and guide future policy development.

## 2. Results

### 2.1. Demographics/Participant Information

The survey was completed by 322 veterinarians, the majority of whom (71%, *n* = 227) worked in general practice. An additional 16% of participants were engaged in other areas of clinical practice, including emergency (3.5%), referral (6.2%), or university practice (6.2%). The remaining participants worked in government (3.1%), industry/pharmaceutical (2.8%) or university teaching and research roles (7.8%). The distribution across the Australian states was generally consistent with the number of veterinarians registered in those states [45,46], although Victoria was slightly over-represented and our survey population also included non-registered veterinarians, about whom there is limited demographic data available. Assuming a maximum population size of veterinarians in Australia of 15,000, our 322 survey respondents yielded a confidence interval of 93%.

Respondents worked in rural (40%) and metropolitan locations (60%), with metropolitan areas defined as a population over 100,000 people. Survey respondents were largely graduates from the last twenty years (61%), but there were respondents from all graduation years between 1971 and 2019. Post-graduate qualifications were common among participants (40%), with membership of the Australian and New Zealand College of Veterinary Science (34%) or specialist qualifications (20%) the most frequently attained. All the recent graduates (classes of 2016–2019) that responded to the survey were in clinical practice. Refer to Supplementary Materials for further information.

### 2.2. Knowledge and Use of Rating Systems

Of the 278 veterinarians working in clinical practice, 49% (*n* = 135) knew of the ASTAG antimicrobial importance rating system, and 22% (*n* = 60) used a traffic light system for antimicrobial importance in their workplace. An example of a traffic light system for antimicrobial importance may include antimicrobials held in the clinic being colour coded in green, orange or red based on their ASTAG importance rating using a sticker or sign on the packaging or the shelf where the antimicrobials are stored. Of the 44 non-clinical veterinarians, 66% (*n* = 29) knew of the ASTAG antimicrobial importance rating system, and 32% (*n* = 14) used a traffic light system for antimicrobial importance in their workplace. Veterinarians with post-graduate qualifications working in clinical practice were 1.7 times (RR: 1.7; 95% CI: 1.4–2.2; *p* < 0.05) more likely to have known of the ASTAG rating system (67%) compared to veterinarians in clinical practice without post-graduate qualifications (39%), but differences in the use of a traffic light system for antimicrobial importance were not significant (RR: 1.25; 95% CI: 0.79–1.97; *p* = 0.36). Recent graduates were more likely to be aware of the ASTAG rating system (62%) compared to more experienced clinicians (46%) (RR: 1.35; 95% CI: 1.0–1.8; *p* = 0.05), but this did not translate into a significant increase in the use of a traffic light system in the workplace (RR: 1.15; 95% CI: 0.7–2.0; *p* = 0.69). There was no difference between rural clinicians and those in metropolitan areas in awareness of the ASTAG rating system (RR: 1.04; 95% CI: 0.8–1.3; *p* = 0.08), or in the use of a traffic light system in their workplace.

### 2.3. Agreement with Restrictions

Overall, when asked whether veterinarians should be able to prescribe antimicrobials with high importance to human medicine *without* restrictions, 61% (*n* = 196) of participants disagreed, while 35% (*n* = 113) agreed that they should. Disagreement was highest for veterinarians not in clinical practice (73%, *n* = 32), followed by recent graduates (65%, *n* = 34) and clinical veterinarians (59%, *n* = 164). Veterinarians with post-graduate qualifications who were working in a clinical role had higher agreement (46%, *n* = 44) that veterinarians should be able to prescribe high-importance antimicrobials without restrictions, whereas only 31% (*n* = 10) of veterinarians with post-graduate qualifications working in a non-clinical role agreed with this proposition.

**Figure 1:**
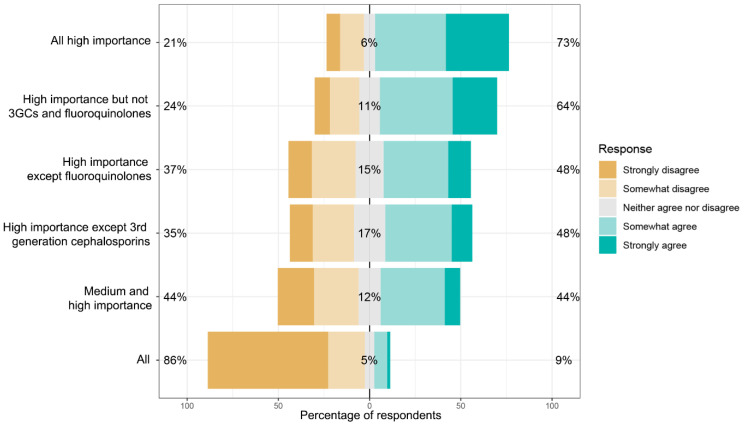
Responses of all participants to the question: “If there were to be restrictions placed on veterinary prescribing of antimicrobials, to which antimicrobials should they apply?”.

When all participants were asked, *‘If there were to be restrictions placed on veterinary prescribing of antimicrobials, which antimicrobials should they apply to?’*, there was most agreement for restricting high-importance antimicrobials (73%), followed by restricting high-importance antimicrobials but excluding third-generation cephalosporins and fluoroquinolones from these restrictions (64%) (Figure 1). There was minimal agreement with restricting all antimicrobials (9%), and this option also elicited the most ‘strongly disagree’ responses (66%). For clinicians, only 6% agreed with restricting all antimicrobials and 89% disagreed or strongly disagreed with this proposition.

More recent graduates (those who had graduated in the years 2016–2019) agreed that restrictions should apply to all high-importance antimicrobials (83%). This was a greater level of agreement than among experienced clinical veterinarians (greater than five years after graduation), 71% of whom agreed that restrictions should apply to all high-importance antimicrobials. Experienced veterinarians who were not working in clinical practice had higher levels of agreement that restrictions should apply to high-importance antimicrobials (76%), and had much lower levels of agreement that restrictions should apply to high-importance antimicrobials but exclude third-generation cephalosporins and fluoroquinolones (42% compared to 68% of experienced clinical veterinarians). Uncertainty around the option of excluding third-generation cephalosporins from restrictions was pronounced among recent graduates (26% neither agreed nor disagreed) and non-clinical veterinarians (25%), whereas only 13% of more experienced veterinarians in clinical practice were uncertain about this option.

**Figure 2:**
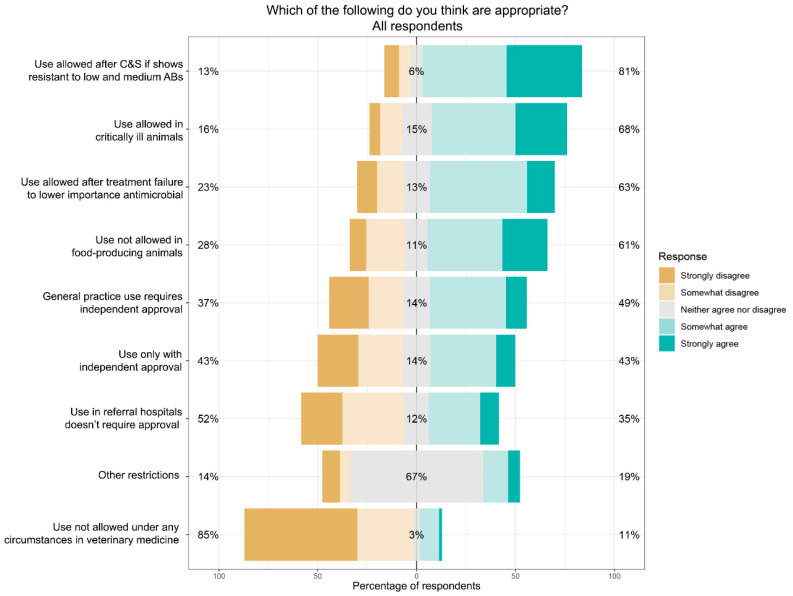
Responses of all participants to the question: “If there were to be restrictions placed on veterinary prescribing of antimicrobials with a high importance rating, which of the following do you think is appropriate?”.

Participants were asked about the appropriateness of various restrictions if they were to be placed on the use of high-importance antimicrobials. Figure 2 shows the responses for all participants. There was most agreement (81%) for use to only be allowed after culture and susceptibility (C&S) testing confirmed that the pathogen was resistant to all low- and medium-rated antimicrobials that could be used to treat the case.

**Figure 3:**
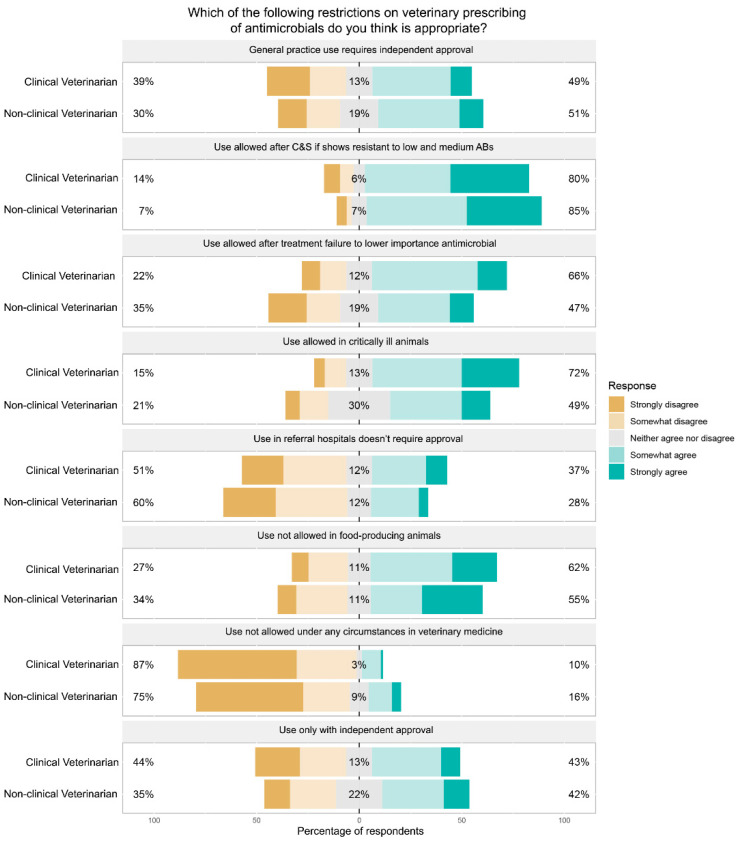
Responses of clinical and non-clinical veterinarians to the question “If there were to be restrictions placed on veterinary prescribing of antimicrobials with high importance rating, which of the following do you think is appropriate?

Support for other restrictions was noticeably different between veterinarians who were and were not working in clinical positions (Figure 3). Clinical veterinarians were more inclined to support use after the failure of treatment with low- and medium-rated antimicrobials (66% agreement compared to 47% agreement amongst non-clinicians). There was also more support amongst clinicians for use to be allowed in critically ill animals (72% agreement compared to 49% amongst non-clinicians). Clinical veterinarians were more likely to agree that the use of high-importance antimicrobials must never be allowed in food-producing animals, but can be allowed in other animals (62% compared to 55% of non-clinical veterinarians). However, 16% of non-clinicians agreed with restricting high-importance antimicrobials so their use was not allowed under any circumstances in veterinary medicine, whereas only 10% of clinicians supported this restriction. Almost half (49%) of veterinarians in clinical practice agreed with the restriction, ‘Use in general practice requires approval from an independent office’ and 13% neither agreed nor disagreed with this restriction.

For the small group of respondents in referral practice (*n* = 20), the most agreement was for use being allowed in critically ill animals without restriction and use being allowed after culture and susceptibility testing confirmed that the pathogen was resistant to all low- and medium-rated antimicrobials that could be used to treat the case (83% in both cases). Fifty percent of referral veterinarians agreed or strongly agreed that use in referral hospitals should not require approval.

Three questions were asked to clarify whether respondents felt that certain conditions should preclude the need for approval (Table 1). If culture and susceptibility testing confirmed that the pathogen was resistant to all low- and medium-rated antimicrobials, most respondents (51%, *n* = 160) indicated that approval should still be required for the use of high-importance drugs.

### 2.4. Case Scenarios and Reasonable Treatments

Participants were provided with several case scenarios and asked whether use was appropriate or not.

**Scenario 1:** *A cat presents with a draining abscess on its face. While the cat is febrile and inappetent and you believe that antimicrobial therapy is indicated, you do not think that the infection is life-threatening. Under which circumstances is it reasonable to treat this cat with cefovecin (long-acting 3rd generation cephalosporin), an antimicrobial with a high importance rating?*

**Figure 4:**
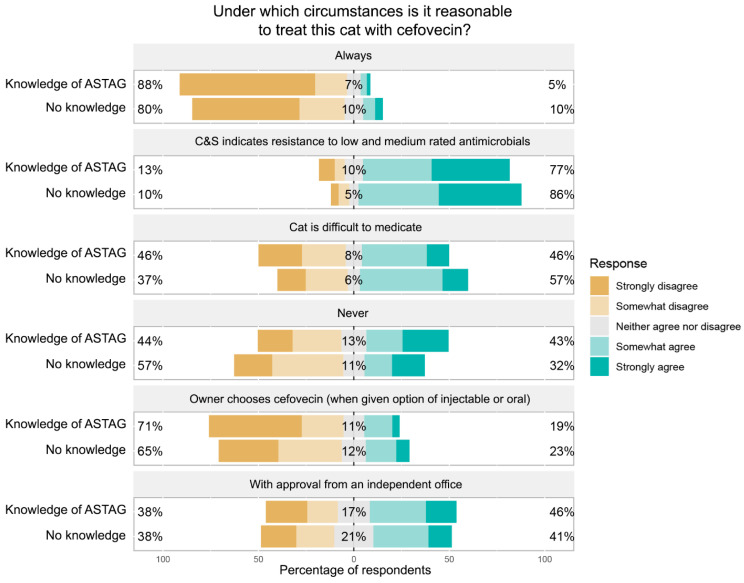
Responses of participants about the reasonableness of use of cefovecin in different circumstances, with responses segregated based on the respondents’ knowledge of the ASTAG rating system.

Veterinarians with knowledge of the ASTAG rating system had lower levels of agreement with the use of cefovecin in all the situations except when approval was given by an independent office (46% veterinarians with knowledge, 41% of those with no knowledge) and with the option of use never being reasonable (43% with knowledge, 32% with no knowledge) (Figure 4). Culture and sensitivity results suggesting resistance to low- and medium-rated antimicrobials was the situation in which the greatest proportion of respondents considered the use of cefovecin to be reasonable.

A greater proportion of clinical veterinarians who started practice after the release of cefovecin onto the Australian market in 2008 agreed that its use for a cat that was difficult to medicate was reasonable (61%) compared to veterinarians who started practice before the release of cefovecin (50%), although this difference was not statistically significant (RR: 0.78; CI: 0.58–1.05; *p* = 0.1).

**Scenario 2:** *A horse presents with a septic fetlock joint that could be life-threatening. Under which circumstances is it reasonable to inject the joint with amikacin, a high-importance rated aminoglycoside?*

**Figure 5:**
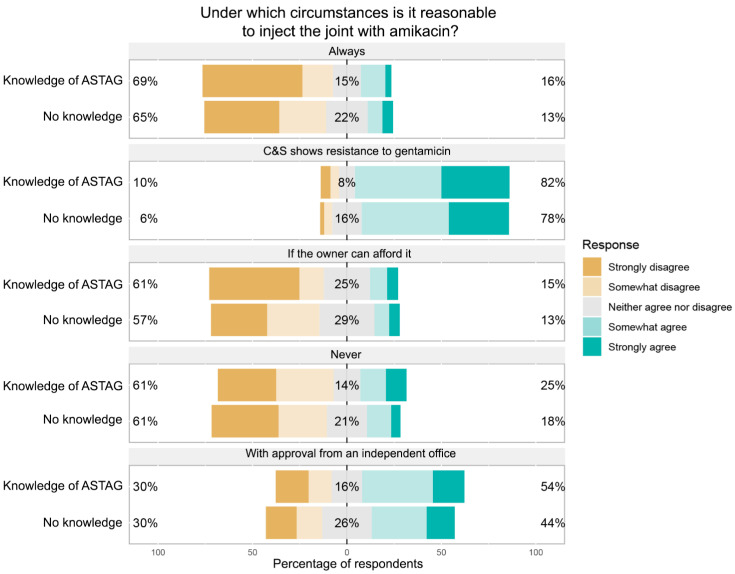
Responses of participants regarding reasonableness of injecting a horse’s fetlock with amikacin in different circumstances, with responses segregated based on the respondents’ knowledge of the ASTAG rating system.

In this scenario, a culture and sensitivity testing result indicating resistance to a medium-importance antimicrobial (gentamicin) was also a situation in which the greatest proportion of respondents agreed that use was reasonable (Figure 5). A higher proportion of respondents with knowledge of the ASTAG rating system thought use was reasonable if it was approved by an independent office (54% agreed or strongly agreed) compared to 44% of those who had not heard of the ASTAG rating system.

Respondents were also asked about several other factors that might influence their decision-making about the horse in Scenario 2. The horse’s insurance status and owner demand were the factors that respondents least often felt ‘should be taken into account’ (Table 2).

Very few respondents thought that ‘any antimicrobial should be allowed’ under either of the scenarios. The situations for which respondents responded with ‘should be taken into account’ or ‘makes some difference’ were if other treatments had failed (82%, *n* = 248) and if the horse was a food-producing species in that jurisdiction (70.1%, *n* = 211).

**Scenario 3:** *A dairy cow presents with pneumonia and you believe antimicrobial therapy is indicated although the infection is not life-threatening. Under which circumstances is it reasonable to treat this cow with ceftiofur (3rd generation cephalosporin), an antimicrobial with a high-importance rating?*

**Figure 6:**
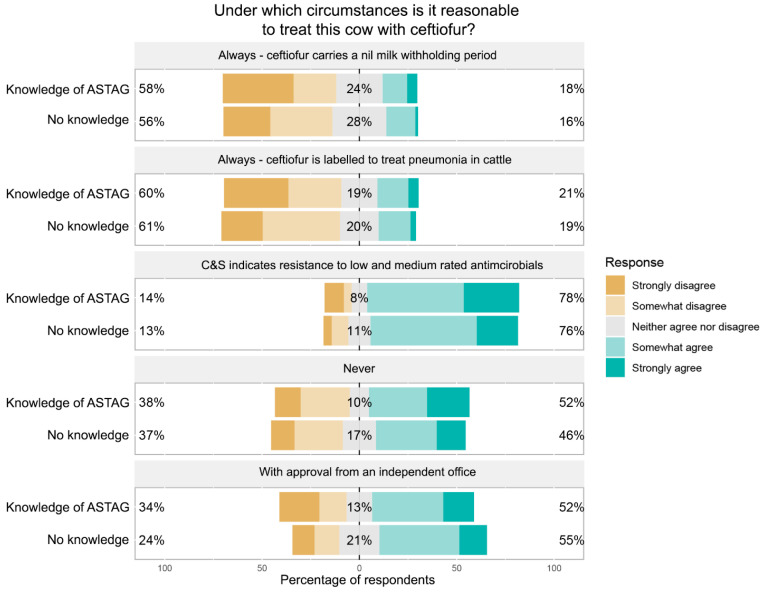
Responses of participants about the reasonableness of treating pneumonia in a cow with ceftiofur, with responses segregated based on the respondents’ knowledge of the ASTAG rating system.

In this scenario, a culture and sensitivity test result indicating resistance to lower-rated antimicrobials was also a situation in which the greatest proportion of respondents agreed that use was reasonable (Figure 6). Of the three scenarios, *Scenario 3*, the only example featuring a food-producing animal, was the scenario in which the highest proportion of veterinarians thought the use of ceftiofur was never appropriate. The labelling of ceftiofur (nil milk withholding period and labelled to treat pneumonia in cattle) was not regarded by most respondents as reasonable grounds for its use in this scenario.

### 2.5. Free-Text Responses

The cost of bacterial C&S was cited as an issue for veterinarians. Cost was an important factor when making prescribing decisions before antimicrobial use, e.g.,

*“…cost of C&S is high for our clients. This also contributes to not ‘working up cases properly’, or even bothering with the negotiations of this with clients.”*

However, comments were made in support of using C&S testing first as a way of deciding on or allowing the use of high-importance antimicrobials, e.g.,

*“Antimicrobials should be split into 2 categories. “Empirical therapy” and “after C&S”. The only restrictions put onto antimicrobials should be that C&S MUST be performed before administering it”.*

The most common free-text comments were about the practicalities of implementing any kind of restriction requiring an external approval system, particularly given the smaller size of veterinary practices compared to human hospitals and when veterinarians were working after hours and on weekends or public holidays.

*“In the case of a critical patient there is definitely no time to waste waiting for approval, not to mention the added time and strain on all ready overworked vets, especially those on call in the middle of the night”*

*“…I would be happy with requiring approval in all cases if approval could be reasonably readily got (or denied) within an hour or two, 24 h a day. If it’s going to take longer then I would prefer to be able to use them on the basis of susceptibility testing showing they were the only reasonable option or (perhaps) in critical patients.*

*“Differences between human and veterinary medicine is the size and integration of the human medical system, which I would anticipate allows for faster approvals of restricted drugs. In veterinary medicine we often see patients presenting with disease courses further along in the disease process and the industry consists of mainly independent small businesses. Applying the same approval requirements would be logistically difficult to achieve in a timely manner for critically ill patients.”*

Concerns for animal welfare were also raised, particularly if there would be delays in obtaining approval to use an antimicrobial when there was a perceived need.

*“Restrictions on high importance antimicrobials are good in theory, as long as in-practice turnaround times for approval does not compromise ability to treat critically ill animals requiring antimicrobials of high importance.”*

Restrictions on prescribing were seen as restricting a veterinarian’s ability to do their work and fulfil other requirements of their role, such as maintaining animal health and welfare.

*“In cattle and horse practice, Excede [ceftiofur] has been used a lot as a long-acting antimicrobial which has high practical value for reduced handling of stock with associated welfare benefits. With horses it can be a way to give a Hendra [virus] exclusion case some antibiotic cover to fulfil WH and S [workplace health and safety laws] and welfare obligations.”*

*I see strong industry effort to reduce the use and to use them more appropriately, and I believe this is key; not restrictions that could potentially harm the patients we seek to protect and heal (because of human medical industries).*

The need for the further education of veterinarians and clear guidance was frequently mentioned as a solution to the issue, either instead of or as well as other restrictions.

*“Mandatory AMS programs in all vet practices would be a good idea. I think we need to prove that we are using them only in situations where there is a lot of thought put into it—high important antimicrobials are important for a reason”*

*“Better education of small animal practitioners required. Vets who work with food producing animals are more aware generally.”*

*“I’m not sure. External approvals create a lot of cost and bureaucracy, but we seem incapable of disciplining ourselves to be scientific in our use of antimicrobials. Maybe looser controls if a practitioner has undergone training and has an antimicrobial stewardship plan in place.”*

*“Clear guidelines for general practitioners on when use of high importance antibiotics may be valid, and equally, when use is not valid, are important.”*

The issue of the off-label use of compounded and human products in veterinary medicine was raised and it was highlighted that these products also need to be included if reporting or auditing of any kind is implemented. The use of combination antimicrobial therapy was also mentioned several times.

*“This reporting MUST include compounding and human products as these are completely under the radar.”*

*“Multiple antibiotic combinations need regulating.”*

Many comments were made in favour of restrictions of some kind on the veterinary prescribing of antimicrobials, particularly high-importance antimicrobials. Many of the comments opposing restrictions raised concerns about the imposition of restrictions on veterinarians who may not be misusing antimicrobials or about limiting veterinarians’ professional right to prescribe antimicrobials. Some respondents suggested they felt they were being unfairly blamed or targeted.

*“I believe the veterinarian, like a human doctor should have the professionalism to decide when they choose to use antibiotics. We are not different to human doctors. I believe I am a professional and I believe I make professional decisions. I do not feel I need to be regulated.”*

*“It is not a good idea to impose further restrictions on those vets that are already doing the right thing.”*

*“I am very concerned about this matter, but I am as concerned by the blatant misuse of antimicrobials in human health and their misuse in human and animal medicine overseas in contrast to Australia. More regulation in our highly regulated country will lead to worsening of animal health and welfare outcomes. Education is key, not more regulation.”*

*“It is important for vets and medicos to work together to lessen our general use of antimicrobials. I resent vets being put to blame for overuse in humans as well.”*

*“I can’t help but feel what we do in a clinic setting will have little overall impact—I think vets in general are judicious…Antibiotic resistance is much more likely to develop from the profligate use and over-the counter access to them in less regulated countries.”*

Some of the comments made in favour of restrictions referred to concerns about the prescribing practices of colleagues.

*“I think we need a lot more regulations as a lot of my colleagues I work with reach for 3rd generation antibiotics as the first antimicrobial to use on the patient almost 90% of the time.”*

*“The use of ceftiofur (especially), enrofloxacin and gentamicin in horse practice and especially racehorse track practice is a joke. Common to give ceftiofur either prophylactically pre-race or even if the horse has minimal symptoms…Needs to be stopped!”*

*“The off-label use of ceftiofur to treat scouring calves or calves with pneumonia, for treating endometritis or for treating lame cows are the scenarios that are far more risky (and more common) than use for treating the odd case of pneumonia and are conditions for which there are alternatives.”*

Some of the suggestions of respondents for improving AMS in the profession included a phone hotline for advice, having prominent warning labels on high-importance antimicrobials and monitoring the use of antimicrobials by veterinarians using post-prescription auditing, particularly for high users.

## 3. Discussion

### 3.1. Most Veterinarians Are Open to Some Restrictions on Antimicrobial Prescribing

The results from this survey suggest that the majority of veterinarians agree with more stringent conditions on prescribing antimicrobials, in particular, those of high importance. This contrasts with the findings of a survey undertaken in 2016, where veterinarians did not think restrictions would be helpful [40]. This could be a consequence of a change in sentiment over time, in line with increasing awareness of the issue of AMR amongst veterinarians and a focus on this in Australian veterinary schools [47,48].

Previous research has identified concerns among professionals about a loss of professional autonomy and feelings of resentment about unwarranted blame if the prescribing of antimicrobials is restricted [40,49]. Some of the free-text comments in our survey also referred to these themes; however, concerns about practicalities and animal welfare were more common. The framing of questions may have influenced the differences between responses to our survey and the survey conducted in 2016. Our question was asked in the context of doctors needing to obtain approval to prescribe high-importance antimicrobials for patients and this not being the case in veterinary practice. This may have appealed to the sense of equality that causes some veterinarians to usually oppose restrictions when questions about restrictions are asked without reference to restrictions on medical prescribing. Given the effect that restrictions on antimicrobial use have had in human medicine, both in Australia and internationally [36,37,50], the education of veterinarians and veterinary students should focus on the benefits of implementing restrictions. These could include better relationships with clients, improved management through more frequent veterinary consultations, and improved clinical care as a result of more accurate diagnoses, as might be the case if diagnostic testing is required prior to prescribing high-importance antimicrobials. Improvements in workplace culture may also be a positive outcome of restrictions if they facilitate discussion amongst colleagues and the adoption of more evidence-based practices. When managing ‘common pool’ resources such as antimicrobials, approaches that foster cooperation and provide a sense of collective ownership of the issue, and the interventions to address it, can be more beneficial than restrictions alone [49].

### 3.2. Most Popular Restrictions and Significance of Culture and Sensitivity Testing

Restricting the prescribing of all high-importance antimicrobials was the restriction with the most support, followed by restricting high-importance antimicrobials but excluding third-generation cephalosporins and fluoroquinolones. As veterinarians, particularly companion animal veterinarians, already prescribe these classes of high-importance antimicrobials, it might be expected that some veterinarians would prefer that they were not restricted in any way.

The most popular restriction was that “Use is only allowed after culture and susceptibility testing confirms the pathogen is resistant to all low- and medium-rated antimicrobials that could be used to treat the case.” This was also a situation in which most respondents felt that the use of high-importance antimicrobials was reasonable in all three case-based scenarios. Respondents were divided on whether additional approval was required on top of C&S results. Table 1 shows that 51% of respondents thought additional approval was still required after C&S testing. The imposition of a requirement for further approval after C&S testing also yielded the highest level of disagreement (37%), suggesting that many respondents thought that use of high-importance antimicrobials should be allowed without any additional approval process if C&S test results indicated that they were the only effective option.

It is possible that responses may depend on which specific high-importance antibiotic agent is being used, rather than high-importance antimicrobials in general, or even particular classes of antibiotics. Our case-based scenarios only referred to two third-generation cephalosporins—cefovecin and ceftiofur—and the high-importance aminoglycoside amikacin, and included limited information about the patient presentation and the available options. Some respondents may have made a different choice in a real clinical scenario. Evidence from human medicine has frequently focused on the restriction of single classes of antimicrobials or, in some cases, restrictions or new procedures for prescribing a single antimicrobial [51,52,53]. Similar targeted research in a veterinary setting will be required over time to build on our study as more veterinary practices implement antimicrobial stewardship strategies.

Where restrictions have been introduced into human hospital settings, and where they have been introduced in animal health settings, they have been shown to have a dramatic effect on the quality of prescribing, in terms of compliance with guidelines or appropriateness, as well as the effects on reducing levels of AMR [12,16,35,53,54]. However, the acceptance of restrictions by prescribers is considered a barrier to antimicrobial stewardship [53,55,56,57]. Most success appears to be achieved when education for healthcare teams is provided alongside the implementation of restrictions. One successful example has been the introduction of pre- or post-prescribing review and feedback, where an infectious diseases physician reviews prescriptions either before they are provided or in the 24–48 h afterwards and gives face-to-face or written feedback to prescribers about their choices [16,35,58,59,60]. Most Australian veterinary practices do not have readily available access to trained staff who could provide pre- or post-prescribing review or feedback. Therefore, the implementation of restrictions combined with other educational support and relevant diagnostic testing (such as C&S testing) is likely to be a more feasible option in terms of time and human resources.

Given the concerns and difficulties veterinarians have reported about the costs of testing to clients (also evident in free-text comments in this study) as well as the practicalities of waiting for C&S results, which can take 2–3 days depending on location, it is interesting that most respondents identified C&S testing as an appropriate restriction on the prescribing of high-importance antimicrobials and agreed with its use in determining the reasonableness of antimicrobial use. If most veterinarians are in favour of using more C&S testing to improve the quality of prescribing and the main barrier is cost, exploring the impacts on antimicrobial prescribing of subsidising or reducing C&S testing costs is an important target for future AMS interventions and should be explored as part of a One Health approach to addressing AMR [61,62]. Improving communication with clients about the benefits of using these diagnostic tests could also be beneficial [31,63,64]

Respondents appeared to have higher thresholds for reasonable use in the production animal scenario than in the equine and feline scenarios. This may be attributable to the strict legislation already in place in Australia around use of antimicrobials in food-producing animals and the awareness of veterinarians about the risk of residues entering the food chain, and about the importance of infection control, biosecurity, vaccination and other herd health interventions for managing disease in farm animals [12,47].

### 3.3. Influence of ASTAG Awareness and Other Participant Characteristics

Those respondents who were aware of the ASTAG rating system were more likely to respond to the clinical scenarios in a way that was consistent with current Australian-specific veterinary antimicrobial use guidelines. This may reflect greater familiarity with prescribing guidelines, more concern about AMR, or a higher level of knowledge about antimicrobial prescribing.

Recent graduates have more recent knowledge and training in antimicrobial stewardship, which was consistent with their higher level of awareness of the ASTAG guidelines. However, this did not necessarily result in a more appropriate approach to antimicrobial use. The pressures of clinical practice and concerns about what owners want, as well as pressure from colleagues, may have a stronger influence on less experienced veterinarians [31,57,64,65]. Other research has shown that veterinarians and human health practitioners are influenced by what they have been taught and the pressures of daily practice, including the behaviour of colleagues, particularly those with more experience or seniority [57,66,67]. Similar factors may have influenced recent graduate veterinarians in this survey, impacting the appropriateness of their choices in spite of their more up-to-date knowledge. For example, 21% of recent graduates agreed that use of amikacin use was reasonable in Scenario 2 if owners could afford it, compared to only 14% of all respondents. Given that this was their response to a survey question, rather than a clinical case with the pressures that might be felt in practice, it is particularly interesting that recent graduates responded differently. The reasons for this should be explored further.

One of the case-based scenarios provoked a higher level of uncertain responses from recent graduates (graduated 2016–2019)—whether use of cefovecin was reasonable in a cat that was difficult to medicate, with 14% neither agreeing nor disagreeing, compared with an uncertain response rate of only 7% amongst practitioners who had graduated prior to 2008, when cefovecin was first registered for use in Australia. Having restrictions in place could be helpful for recent graduates in such situations, particularly if restrictions are implemented in concert with further education and the use of guidelines. This might be expected to facilitate a sense of collective ownership of the problem and solutions, rather than an expectation that it is more appropriate to defer to what other veterinarians in the practice are doing even if this is not guideline-compliant [49]. Free-text comments on this theme stated,

*“Other than reserving some antimicrobials to human use only, there should be clear guidelines that we can point clients to when they get frustrated.”*

*“I think high importance antimicrobials are often used because inexperienced practitioners lack confidence in themselves.”*

While experienced clinical veterinarians are less impacted by financial concerns and client demands (real or perceived), they may also be more likely to rely on their past experience rather than on guidelines [31,65,68]. This was seen in a survey of Australian dairy cattle veterinarians, where clinical experience was the most influential factor affecting prescribing choices, followed by product label recommendations [69]. In that same survey of dairy cattle veterinarians, the likelihood of choosing a first-line treatment for mastitis via dry cow therapy decreased with the number of years of experience.

The impact of post-graduate qualifications on agreement with restrictions may have differed between those veterinarians who were in clinical and non-clinical roles for several reasons. Those in clinical practice with post-graduate qualifications are likely to be specialists and have significant experience in their field. This can lead to reliance on past experience and a greater trust in one’s own knowledge, rather than feeling that restrictions are required to improve the quality of prescribing [57].

Non-clinical veterinarians were generally more in favour of restrictions, which may be related to their separation from the daily challenges of clinical practice that influence prescribing choices. The pressures and practicalities of clinical practice and treating ill animals may also explain why clinical veterinarians were more inclined to support the use of high-importance antimicrobials after the failure of treatment with low- and medium-rated antimicrobials, and why there was significantly more support amongst clinicians for the use of high-importance antimicrobials in critically ill animals. Respondents in specialist referral practice are likely to see more severe and complex cases, and thus, might be expected to believe that use in critically ill animals should not be subject to restriction.

Unlike in human healthcare, practice in rural or metropolitan areas did not seem to influence prescribing significantly in our survey, a finding that is consistent with other veterinary research [31]. This could be because the difference between rural and metropolitan veterinary practice is less distinct, because veterinary clinics in regional locations are often better equipped and have greater in-house diagnostic capacity than smaller metropolitan practices, which can be more reliant on specialist, referral and emergency centres for advanced testing and complex case management.

### 3.4. Issues about Feasibility of Implementing Restrictions

While the responses to some survey questions indicated that respondents did not place as much weight on factors such as owner demand and the insurance status of patients (Table 2), the free-text responses highlighted numerous issues around the feasibility of the implementation of restrictions. These included time pressures on prescribers, concerns about patient deterioration and animal welfare without antimicrobial use, concerns about client perceptions or direct requests for antimicrobials, and costs associated with additional testing. Communication techniques for use by veterinarians when not prescribing antimicrobials have been developed, but have not yet been evaluated [63]. Other issues around feasibility are likely to require increased education for veterinarians and other associated personnel and the development of systems that do not result in additional time pressures for veterinarians, but instead, enable them to obtain permission or support for the appropriate use of high-importance antimicrobials easily when required.

Our survey responses suggest that adopting a non-punitive approach to AMS interventions, including any kinds of restrictions, could help raise awareness of the importance of AMS while also fulfilling the need for increased education about appropriate antimicrobial use and allaying concerns about the practicalities of an approval system. It is clear from the responses to this survey that C&S testing needs to be a key factor in any approval process. However, previous work has highlighted that disease investigation can be difficult in private veterinary practice because there is no or minimal incentive for clients to fund investigations that are primarily in the public interest [70]. To ensure equitable and increased access, public funding for approval systems and for diagnostic testing is justified, given the One Health benefits of addressing AMR [71]. Allocating funding for subsidised veterinary C&S testing may be more cost effective in the long term, as it could preserve the efficacy of antimicrobials for human treatment, particularly in the case of high-importance antimicrobials.

Future research should focus on the practicalities of implementing restrictions and the outcomes of other AMS interventions, such as education programs and subsidised C&S testing. Australia’s National Antimicrobial Resistance Strategy states that “Integrated Surveillance and Response to Resistance and Usage” is one of its objectives, and financial support for veterinary AMS at a federal level through subsidised C&S testing would be very much in keeping with this objective [18,61,72,73]. This would particularly be the case if such a scheme could be used to increase surveillance for AMR in animals.

### 3.5. Other Challenges

A proportion of veterinarians (19%) use a traffic light system for indicating antimicrobial importance in their workplace and 49% were familiar with the ASTAG rating system. This is already a small improvement from a study conducted in 2017, which found that 15% of survey respondents had antimicrobial prescribing policies or AMS policies in their practices. Guidelines were used by 28% of respondents to this 2017 survey. Global initiatives such as Antibiotic/Antimicrobial Awareness Week started in 2015, and an understanding of antimicrobial stewardship amongst veterinarians may have become more common over the past five to ten years, in line with an exponential increase in publications on this topic and the release of Australia’s first National Antimicrobial Resistance Strategy in 2015 [73,74,75]. Ongoing research to track improvements in awareness about AMR and AMS, and guideline use, amongst veterinarians would be beneficial to measure the success of any AMS initiatives. Practical education on how to implement a traffic light system in the workplace has been introduced to some veterinary practices in recent years, and a further focus on this in education for veterinarians, particularly those in senior or management roles in the practice, may help more veterinarians implement this in daily practice. Recent graduates, who have higher awareness of the traffic light system, may also be more likely to use it in practice if it is already an established part of the practices in which they work than if they were responsible for its implementation.

The off-label use of high-importance antimicrobials was highlighted as a concern numerous times in free-text comments in our survey, particularly in relation to dairy cattle and equine practice. Concerns about use of compounded antimicrobials and their bioavailability were also raised. Any restrictions must encompass off-label use, but it is important to realise that off-label use can actually be more appropriate than following product labels in some cases, particularly in relation to dose rates [76,77]. Label warnings on high-importance antimicrobials were suggested and could be an important first step. The label for the third-generation cephalosporin, cefovecin, already states, ‘FOR USE ONLY in dogs and cats where indicated by antibiotic sensitivity testing according to principles of prudent use’ [78]; however, 10% of respondents who were unaware of the ASTAG rating system regarded the use of this antimicrobial for a cat bite abscess without culture and sensitivity to always be reasonable, and 57% of these respondents also thought it was reasonable if the cat was difficult to medicate orally. The widespread use of cefovecin without culture and sensitivity testing has been reported in other studies in Australia and overseas [30,79,80]. While label restraints are important, current evidence suggests they do not seem to have much impact on prescribing by companion animal veterinarians.

## 4. Materials and Methods

The source population for the survey was veterinarians in Australia in 2019/2020. At that time, there were between 12,769 and 13,993 registered veterinarians in Australia; however, the survey was also open to veterinarians working in a non-clinical setting, such as industry, government or academia, who may not currently be registered with their state veterinary surgeons board [45,46]. Respondents self-selected and were invited to participate through email, veterinary organisation newsletters and social media from June 2019 until December 2020. The survey was solely distributed online using the Qualtrics online survey tool. The full survey is available in the Supplementary Materials. The survey questions covered restrictions on the veterinary prescribing of high-importance antimicrobials, the use of antimicrobial importance rating systems and three case scenarios assessing the reasonableness of use of high-importance antimicrobials.

To be confident that the proportion of veterinarians who had heard of the ASTAG rating system was within 7% of a true prevalence of 50%, a total of 194 completed surveys were required. Sample size calculations were carried out with finite population correction, assuming 50% prevalence, because this provided the largest sample size estimate for a constant margin of error. Sample size calculations were made using the samplingbook package in R and the sample.size.prop function. R Core Team (2020). R: A language and environment for statistical computing. R Foundation for Statistical Computing, Vienna, Austria. URL https://www.R-project.org/. Version 1.4.1103 2009–2021.

Short comments in the free-text response options were collated and analysed for emerging themes using qualitative analysis techniques.

## 5. Conclusions

This study has highlighted the need for further targeted research to provide guidance about how restrictions on the use of high-importance antimicrobials should be implemented in veterinary practice. Supporting veterinarians through practical policies and AMS interventions is key if the efficacy of antimicrobials is to be preserved for the treatment of both humans and animals. Education and a non-punitive approach must be integral into any restrictions placed on the prescribing of antimicrobials by veterinarians, who are, in general, open to restrictions and improving the quality of their prescribing. The use and regulation of antimicrobials in veterinary medicine to combat AMR cannot be separated from the manifold challenges facing veterinarians now and in the future. This includes the effects of emerging infectious diseases, including the COVID-19 pandemic, climate change impacts, and workforce challenges. Strategies that are holistic and provide a sense of ownership and empowerment are likely to be the most successful.

## Figures and Tables

**Table 1:** Scenarios requiring approval—responses of all participants.

Question: *Approval is Still Required Prior to High* *Importance Drugs Being Used in Veterinary Medicine*…	Agree (%)	Disagree (%)	Unsure (%)
*…if culture and susceptibility testing confirms that the pathogen is resistant to all low- and medium-rated antimicrobials that could be used to treat the case*	51	37	13
*…if treatment has failed with a lower importance rating antimicrobial*	45	28	26
*… in critically ill animals*	34	33	34

**Table 2:** How would the following situations affect your decision about the horse?

*Situation*	Should Be Taken into Account (%)	Makes Some Difference (%)	Makes no Difference (%)	Any Antimicrobial Should Be Allowed (%)	Unsure (%)
*Other treatments have failed*	166 (55)	82 (27)	26 (9)	10 (3)	(17) 6
*You practice in a region where horses are considered a food-producing species*	163 (54)	48 (16)	42 (14)	2 (1)	46 (15)
*The prognosis was very poor*	132 (44)	79 (26)	63 (21)	6 (2)	21 (7)
*If the horse was the leading thoroughbred stallion in Australia*	76 (25)	59 (20)	128 (43)	6 (2)	33 (11)
*The horse is insured*	33 (11)	32 (11)	202 (67)	3 (1)	31 (10)
*The owners demand amikacin*	16 (5)	37 (12)	218 (72)	2 (1)	28 (9)

## Data Availability

Data is available upon request to the corresponding author.

## Supplementary Materials

The following supporting information can be downloaded at: https://www.mdpi.com/article/10.3390/antibiotics11111589/s1, Document S1: Veterinarian survey—table of participant characteristics, Document S2: Survey instrument.

## References

[1] O’Neill J. (2016). Tackling Drug-Resistant Infections Globally: Final Report and Recommendations—The Review on Antimicrobial Resistance.

[2] Centre for Disease Control (2019). Antibiotic Resistance Threats in the United States 2019.

[3] Millar M. (2011). Can antibiotic use be both just and sustainable... or only more or less so?. J. Med. Ethics.

[4] Wang H., Paulson K.R., Pease S.A., Watson S., Comfort H., Zheng P., Aravkin A.Y., Bisignano C., Barber R.M., Alam T. (2022). Estimating excess mortality due to the COVID-19 pandemic: A systematic analysis of COVID-19-related mortality, 2020–2021. Lancet.

[5] World Health Organization (2021). Global Antimicrobial Resistance and Use Surveillance System (GLASS) Report: 2021.

[6] Tang K.L., Caffrey N.P., Nóbrega D.B., Cork S.C., Ronksley P.E., Barkema H.W., Polachek A.J., Ganshorn H., Sharma N., Kellner J.D. (2017). Restricting the use of antibiotics in food-producing animals and its associations with antibiotic resistance in food-producing animals and human beings: A systematic review and meta-analysis. Lancet Planet. Health.

[7] Brealey J.C., Leitão H.G., Hofstede T., Kalthoff D.C., Guschanski K. (2021). The oral microbiota of wild bears in Sweden reflects the history of antibiotic use by humans. Curr. Biol..

[8] Australian Strategic and Technical Advisory Group on Antimicrobial Resistance (2018). Importance Ratings and Summary of Antibacterial Uses in Humans in Australia.

[9] Australian Pesticides and Veterinary Medicines Authority Substances not Permitted for Use on Food-Producing Animals in Australia. https://apvma.gov.au/node/11626.

[10] Australian Pesticides and Veterinary Medicines Authority (2017). Antibiotic Resistance in Animals.

[11] Jordan D. Antimicrobial Resistance in Food Animals—National and International Developments. https://www.flockandherd.net.au/other/reader/antimicrobial-resistance.html.

[12] Cheng A.C., Turnidge J., Collignon P., Looke D., Barton M., Gottlieb T. (2012). Control of fluoroquinolone resistance through successful regulation, Australia. J. Emerg. Infect. Dis..

[13] Department of Agriculture, Water and the Environment. National Residue Survey. https://www.agriculture.gov.au/ag-farm-food/food/nrs.

[14] Barlow R.S., McMillan K.E., Duffy L.L., Fegan N., Jordan D., Mellor G.E. (2015). Prevalence and Antimicrobial Resistance of Salmonella and Escherichia coli from Australian Cattle Populations at Slaughter. J. Food Prot..

[15] Abraham S., Groves M.D., Trott D.J., Chapman T.A., Turner B., Hornitzky M., Jordan D. (2014). Salmonella enterica isolated from infections in Australian livestock remain susceptible to critical antimicrobials. Int. J. Antimicrob. Agents.

[16] Claeys K.C., Hopkins T.L., Vega A.D., Heil E.L. (2018). Fluoroquinolone restriction as an effective antimicrobial stewardship intervention. Curr. Infect. Dis. Rep..

[17] Unicomb L., Ferguson J., Riley T.V., Collignon P. (2003). Fluoroquinolone resistance in campylobacter absent from isolates, Australia. Emerg. Infect. Dis. J..

[18] Laxminarayan R., Duse A., Wattal C., Zaidi A.K., Wertheim H.F., Sumpradit N., Vlieghe E., Hara G.L., Gould I.M., Goossens H. (2013). Antibiotic resistance—The need for global solutions. Lancet Infect. Dis..

[19] Jiang X.P., Yang H., Dettman B., Doyle M.P. (2006). Analysis of fecal microbial flora for antibiotic resistance in ceftiofur-treated calves. Foodborne Pathog. Dis..

[20] The Joint Expert Technical Advisory Committee on Antibiotic Resistance, Department of Health and Aged Care, Department of Agriculture, Fisheries and Forestry (1999). The Use of Antibiotics in Food-Producing Animals: Antibiotic-Resistant Bacteria in Animals and Humans.

[21] Dutil L., Irwin R., Finley R., Ng L.K., Avery B., Boerlin P., Bourgault A.-M., Cole L., Daignault D., Desruisseau A. (2010). Ceftiofur resistance in Salmonella enterica serovar Heidelberg from chicken meat and humans, Canada. Emerg. Infect. Dis..

[22] Sparham S.J., Kwong J.C., Valcanis M., Easton M., Trott D.J., Seemann T., Stinear T.P., Howden B.P. (2017). Emergence of multidrug resistance in locally-acquired human infections with Salmonella Typhimurium in Australia owing to a new clade harbouring blaCTX-M-9. Int. J. Antimicrob. Agents.

[23] Vieira A.R., Collignon P., Aarestrup F.M., McEwen S.A., Hendriksen R.S., Hald T., Wegener H.C. (2011). Association between antimicrobial resistance in Escherichia coli isolates from food animals and blood stream isolates from humans in Europe: An ecological study. Foodborne Pathog. Dis..

[24] Kieke A.L., Borchardt M.A., Kieke B.A., Spencer S.K., Vandermause M.F., Smith K.E., Jawahir S.L., Belongia E.A. (2006). Use of streptogramin growth promoters in poultry and isolation of streptogramin-resistant Enterococcus faecium from humans. J. Infect. Dis..

[25] Shaban R.Z., Simon G.I., Trott D.J., Turnidge J., Jordan D., Department of Agriculture (2014). Surveillance and Reporting of Antimicrobial Resistance and Antibiotic Usage in Animals and Agriculture in Australia.

[26] Hardefeldt L.Y., Browning G.F., Thursky K.A., Gilkerson J.R., Billman-Jacobe H., Stevenson M.A., Bailey K.E. (2017). Cross-sectional study of antimicrobials used for surgical prophylaxis by bovine veterinary practitioners in Australia. Vet. Rec..

[27] Hardefeldt L.Y., Browning G.F., Thursky K., Gilkerson J.R., Billman-Jacobe H., Stevenson M.A., Bailey K.E. (2018). Antimicrobials used for surgical prophylaxis by equine veterinary practitioners in Australia. Equine Vet. J..

[28] Hur B.A., Hardefeldt L.Y., Verspoor K.M., Baldwin T., Gilkerson J.R. (2020). Describing the antimicrobial usage patterns of companion animal veterinary practices; free text analysis of more than 4.4 million consultation records. PLoS ONE.

[29] Hardefeldt L.Y., Selinger J., Stevenson M.A., Gilkerson J.R., Crabb H., Billman-Jacobe H., Thursky K., Bailey K.E., Awad M., Browning G.F. (2018). Population wide assessment of antimicrobial use in dogs and cats using a novel data source—A cohort study using pet insurance data. Vet. Microbiol..

[30] Hardefeldt L., Hur B., Verspoor K., Baldwin T., Bailey K.E., Scarborough R., Richards S., Billman-Jacobe H., Browning G.F., Gilkerson J. (2020). Use of cefovecin in dogs and cats attending first-opinion veterinary practices in Australia. Vet. Rec..

[31] Hardefeldt L.Y., Gilkerson J.R., Billman-Jacobe H., Stevenson M.A., Thursky K., Bailey K.E., Browning G.F. (2018). Barriers to and enablers of implementing antimicrobial stewardship programs in veterinary practices. J. Vet. Intern. Med..

[32] Hardefeldt L.Y., Browning G.F., Thursky K., Gilkerson J.R., Billman-Jacobe H., Stevenson M.A., Bailey K.E. (2017). Antimicrobials used for surgical prophylaxis by companion animal veterinarians in Australia. Vet. Microbiol..

[33] Hardefeldt L.Y., Holloway S., Trott D.J., Shipstone M., Barrs V.R., Malik R., Burrows M., Armstrong S., Browning G.F., Stevenson M. (2017). Antimicrobial prescribing in dogs and cats in Australia: Results of the Australasian infectious disease advisory panel survey. J. Vet. Intern. Med..

[34] Chatzopoulou M., Reynolds L.J. (2020). Role of antimicrobial restrictions in bacterial resistance control: A systematic literature review. J. Hosp. Infect..

[35] Pitiriga V., Vrioni G., Saroglou G., Tsakris A. (2017). The impact of antibiotic stewardship programs in combating quinolone resistance: A systematic review and recommendations for more efficient interventions. Adv. Ther..

[36] Aarestrup F.M., Seyfarth A.M., Emborg H.D., Pedersen K., Hendriksen R.S., Bager F. (2001). Effect of abolishment of the use of antimicrobial agents for growth promotion on occurrence of antimicrobial resistance in fecal enterococci from food animals in Denmark. Antimicrob. Agents Chemother..

[37] Agersø Y., Aarestrup F.M. (2012). Voluntary ban on cephalosporin use in Danish pig production has effectively reduced extended-spectrum cephalosporinase-producing Escherichia coli in slaughter pigs. J. Antimicrob. Chemother..

[38] Li W., Hou M., Liu C., Xiong W., Zeng Z. (2019). Dramatic decrease in colistin resistance in Escherichia coli from a typical pig farm following restriction of colistin use in China. Int. J. Antimicrob. Agents.

[39] Nobrega D.B., Tang K.L., Caffrey N.P., De Buck J., Cork S.C., Ronksley P.E., Polachek A.J., Ganshorn H., Sharma N., Kastelic J.P. (2021). Prevalence of antimicrobial resistance genes and its association with restricted antimicrobial use in food-producing animals: A systematic review and meta-analysis. J. Antimicrob. Chemother..

[40] Zhuo A., Labbate M., Norris J.M., Gilbert G.L., Ward M.P., Bajorek B.V., Degeling C., Rowbotham S.J., Dawson A., Nguyen K.-A. (2018). Opportunities and challenges to improving antibiotic prescribing practices through a One Health approach: Results of a comparative survey of doctors, dentists and veterinarians in Australia. BMJ Open.

[41] Belay D.G., Jensen J.D. (2020). ‘The scarlet letters’: Information disclosure and self-regulation: Evidence from antibiotic use in Denmark. J. Environ. Econ. Manag..

[42] Maron D.F., Smith T.J.S., Nachman K.E. (2013). Restrictions on antimicrobial use in food animal production: An international regulatory and economic survey. Glob. Health.

[43] More S.J. (2020). European perspectives on efforts to reduce antimicrobial usage in food animal production. Ir. Vet. J..

[44] Rodrigues Da Costa M., Diana A. (2022). A Systematic Review on the Link between Animal Welfare and Antimicrobial Use in Captive Animals. Animals.

[45] Australian Veterinary Association (2019). Australian Veterinary Workforce Survey 2018.

[46] Australian Veterinary Association (2022). Veterinary Workforce Survey 2021.

[47] Hardefeldt L., Nielsen T., Crabb H., Gilkerson J., Squires R., Heller J., Sharp C., Cobbold R., Norris J., Browning G. (2018). Veterinary students’ knowledge and perceptions about antimicrobial stewardship and biosecurity-a national survey. Antibiotics.

[48] McClelland J.W., Norris J.M., Dominey-Howes D., Govendir M. (2022). Knowledge and perceptions of Australian postgraduate veterinary students prior to formal education of antimicrobial use and antimicrobial resistance. One Health.

[49] Tarrant C., Colman A.M., Chattoe-Brown E., Jenkins D.R., Mehtar S., Perera N., Krockow E.M. (2019). Optimizing antibiotic prescribing: Collective approaches to managing a common-pool resource. Clin. Microbiol. Infect..

[50] Kurita G., Tsuyuki Y., Murata Y., Takahashi T., Vet Infection Control Assoc (2019). Reduced rates of antimicrobial resistance in Staphylococcus intermedius group and Escherichia coli isolated from diseased companion animals in an animal hospital after restriction of antimicrobial use. J. Infect. Chemother..

[51] Yarrington M.E., Wrenn R.H., Spivey J., Shoff C.J., Maziarz E.K., Spires S.S., Turner N.A., Diez A., Anderson D.J., Moehring R.W. (2022). Well-being tradeoffs: Effect of loosening overnight restrictions on antimicrobial starts. Infect. Control. Hosp. Epidemiol..

[52] Chin J., Green S.B., McKamey L.J., Gooch M.D., Chapin R.W., Gould A.P., Milliken S.F., Blanchette L.M. (2019). Restriction-free antimicrobial stewardship initiative targeting fluoroquinolone reduction across a regional health-system. Infect. Prev. Pract..

[53] Reed E.E., Stevenson K.B., West J.E., Bauer K.A., Goff D.A. (2013). Impact of formulary restriction with prior authorization by an antimicrobial stewardship program. Virulence.

[54] Moerer M., Merle R., Bäumer W. (2022). A cross-sectional study of veterinarians in Germany on the impact of the TÄHAV amendment 2018 on antimicrobial use and development of antimicrobial resistance in dogs and cats. Antibiotics.

[55] Adekanye U.O., Ekiri A.B., Galipo E., Muhammad A.B., Mateus A., La Ragione R.M., Wakawa A., Armson B., Mijten E., Alafiatayo R. (2020). Knowledge, attitudes and practices of veterinarians towards antimicrobial resistance and stewardship in Nigeria. Antibiotics.

[56] Avent M.L., Hall L., Davis L., Allen M., Roberts J.A., Unwin S., McIntosh K.A., Thursky K., Buising K., Paterson D.L. (2014). Antimicrobial stewardship activities: A survey of Queensland hospitals. Aust. Health Rev..

[57] Ierano C., Rajkhowa A., Peel T., Marshall C., Ayton D., Thursky K. (2020). Antibiotic prescribing in surgery: A clinically and socially complex problem in Australia. Infect. Dis. Health.

[58] Nadrah K., Pirs M., Kreft S., Mueller Premru M., Beovic B. (2018). Impact of cephalosporin restriction on incidence of infections with extended-spectrum beta-lactamase-producing Klebsiella pneumoniae in an endemic setting. J. Chemother..

[59] Hamilton K.W., Gerber J.S., Moehring R., Anderson D.J., Calderwood M.S., Han J.H., Mehta J.M., Pollack L.A., Zaoutis T., Srinivasan A. (2015). Point-of-prescription interventions to improve antimicrobial stewardship. Clin. Infect. Dis..

[60] Van Dort B.A., Penm J., Ritchie A., Baysari M.T. (2022). The impact of digital interventions on antimicrobial stewardship in hospitals: A qualitative synthesis of systematic reviews. J. Antimicrob. Chemother..

[61] Collignon P., McEwen S. (2019). One Health—Its Importance in Helping to Better Control Antimicrobial Resistance. Trop. Med. Infect. Dis..

[62] McEwen S.A., Collignon P.J. (2018). Antimicrobial Resistance: A One Health Perspective. Microbiol. Spectr..

[63] Scarborough R., Hardefeldt L., Browning G., Bailey K. (2021). Pet Owners and Antibiotics: Knowledge, Opinions, Expectations, and Communication Preferences. Antibiotics.

[64] Smith M., King C., Davis M., Dickson A., Park J., Smith F., Currie K., Flowers P. (2018). Pet owner and vet interactions: Exploring the drivers of AMR. Antimicrob. Resist. Infect. Control..

[65] Mateus A.L., Brodbelt D.C., Barber N., Stark K.D. (2014). Qualitative study of factors associated with antimicrobial usage in seven small animal veterinary practices in the UK. Prev Vet. Med..

[66] Doidge C., Hudson C., Lovatt F., Kaler J. (2019). To prescribe or not to prescribe? A factorial survey to explore veterinarians’ decision making when prescribing antimicrobials to sheep and beef farmers in the UK. PLoS ONE.

[67] Lewis P.J., Tully M.P. (2009). Uncomfortable prescribing decisions in hospitals: The impact of teamwork. J. R. Soc. Med..

[68] King C., Smith M., Currie K., Dickson A., Smith F., Davis M., Flowers P. (2018). Exploring the behavioural drivers of veterinary surgeon antibiotic prescribing: A qualitative study of companion animal veterinary surgeons in the UK. BMC Vet. Res..

[69] Tree M., McDougall S., Beggs D.S., Robertson I.D., Lam T.J.G.M., Aleri J.W. (2022). Antimicrobial use on Australian dairy cattle farms—A survey of veterinarians. Prev. Vet. Med..

[70] Steele S.G., Mor S.M., Toribio J.L.M.L. (2021). ‘It’s our job’: Constraints to investigation of atypical disease events—Opinions of Australian veterinarians. Zoonoses Public Health.

[71] Robinson T.P., Bu D.P., Carrique-Mas J., Fèvre E.M., Gilbert M., Grace D., Hay S.I., Jiwakanon J., Kakkar M., Kariuki S. (2016). Antibiotic resistance is the quintessential One Health issue. Trans. R. Soc. Trop. Med. Hyg..

[72] Djordjevic S., Morgan B.S. (2019). A One Health genomic approach to antimicrobial resistance is essential for generating relevant data for a holistic assessment of the biggest threat to public health. Microbiol. Aust..

[73] Department of Health, Commonwealth of Australia (2019). Australia’s National Antimicrobial Resistance Strategy–2020 and Beyond.

[74] Sweileh W.M. (2021). Bibliometric analysis of peer-reviewed literature on antimicrobial stewardship from 1990 to 2019. Glob. Health.

[75] Commonwealth of Australia (2021). Final Progress Report—Australia’s First National Antimicrobial Resistance Strategy 2015–2019.

[76] Hardefeldt L.Y., Gilkerson J.R., Billman-Jacobe H., Stevenson M.A., Thursky K., Browning G.F., Bailey K.E. (2018). Antimicrobial labelling in Australia: A threat to antimicrobial stewardship?. Aust. Vet. J..

[77] Hardefeldt L.Y., Bailey K.E., Slater J. (2020). Overview of the use of antimicrobial drugs for the treatment of bacterial infections in horses. Equine Vet. Educ..

[78] Australian Pesticides and Veterinary Medicines Authority Public Chemical Registration Information System Search—Convenia. https://websvr.infopest.com.au/LabelRouter?LabelType=L&Mode=1&ProductCode=60461.

[79] Hubbuch A., Schmitt K., Lehner C., Hartnack S., Schuller S., Schupbach-Regula G., Mevissen M., Peter R., Muntener C., Naegeli H. (2020). Antimicrobial prescriptions in cats in Switzerland before and after the introduction of an online antimicrobial stewardship tool. BMC Vet. Res..

[80] Burke S., Black V., Sanchez-Vizcaino F., Radford A., Hibbert A., Tasker S. (2017). Use of cefovecin in a UK population of cats attending first-opinion practices as recorded in electronic health records. J. Feline Med. Surg..

