# Supplementary material for: Attitudes towards Use of High-Importance Antimicrobials—A Cross-Sectional Study of Australian Veterinarians
Source: Antibiotics (Basel). 2022 Nov 10;11(11):1589. doi: 10.3390/antibiotics11111589 (PMC9686791; doi:10.3390/antibiotics11111589)
Supplement: Supplementary file 1 [file antibiotics-11-01589-s001.zip › Document S1.pdf]

Participant Characteristics

Work Location		State/Territory	
Rural	128 (39.9)	Australian Capital Territory	6 (1.9)
Metropolitan	193 (60.1)	New South Wales	84 (26.2)
		Northern Territory	3 (0.9)
**Gender**		Queensland	51 (15.9)
Male	98 (30.9)	South Australia	28 (8.8)
Female	216 (68.1)	Tasmania	6 (1.9)
Rather not say	3 (0.9)	Victoria	114 (35.6)
		Western Australia	28 (8.8)
**Work Type**		**Position**	
First opinion/general	227 (70.5)	Partner	85 (26.4)
Emergency	11 (3.4)	Associate	153 (47.5)
Referral	20 (6.2)	Locum/casual	26 (8.1)
University - teaching & research	25 (7.8)	Other	58 (18)
University - clinical	20 (6.2)	**Veterinarians in workplace**	
Government	10 (3.1)	1 or 2	68 (21.1)
Industry/pharmaceutical	9 (2.8)	3 or 4	77 (23.9)
**Post Graduate Qualification**		5-Oct	89 (27.6)
Yes	128 (39.8)	Nov-20	36 (11.2)
Membership	66 (34.2)	>20	52 (16.1)
Specialist	39 (20.2)	**Graduation Year**	
Masters	34 (17.6)	1971-1980	19 (5.9)
Other	33 (17.1)	1981-1990	54 (16.9)
PhD	21 (10.9)	1991-2000	51 (15.9)
No	194 (60.2)	2001-2010	95 (29.7)
		2010-2020	101 (31.6)
