# Supplementary material for: Attitudes towards Use of High-Importance Antimicrobials—A Cross-Sectional Study of Australian Veterinarians
Source: Antibiotics (Basel). 2022 Nov 10;11(11):1589. doi: 10.3390/antibiotics11111589 (PMC9686791; doi:10.3390/antibiotics11111589)
Supplement: Supplementary file 1 [file antibiotics-11-01589-s001.zip › Document S2.pdf]

Use of Antimicrobials with High Importance to Human Medicine by Vets

Click on the link for more information including ethics approval

Information about the survey and ethics

[Click here for information about the survey and ethics]

INFORMATION ABOUT YOU

What type of veterinary work are you currently employed to do?

- First opinion/general
- Emergency
- Referral
- University - teaching & research
- University - clinical
- Government
- Industry/pharmaceutical

Where is your workplace located?

- Metropolitan (population >100,000)
- Rural

How many veterinarians are employed in your workplace?

- 1 or 2
- 3 or 4
- 5-10

- 11-20
- >20

In which state do you practice?

- ACT
- NSW
- NT
- SA
- QLD
- TAS
- VIC
- WA

Gender

- Male
- Female
- Non-binary
- Rather not say

What is your position in the workplace?

- Partner
- Associate
- Locum/casual
- Other

What year did you graduate?

Do you hold post-graduate qualifications in veterinary medicine?

- ◯ Yes
- ◯ No

What qualifications do you have?

- ☐ Membership
- ☐ Masters
- ☐ Certificate
- ☐ Specialist (Fellowship or Diplomate)
- ☐ Diploma
- ☐ PhD
- ☐ Other

Antimicrobials with high importance for human medicine in Australia are classified by the Australian Strategic and Technical Advisory Group on AMR (ASTAG). Here's an example: You can also access the classifications at this link.

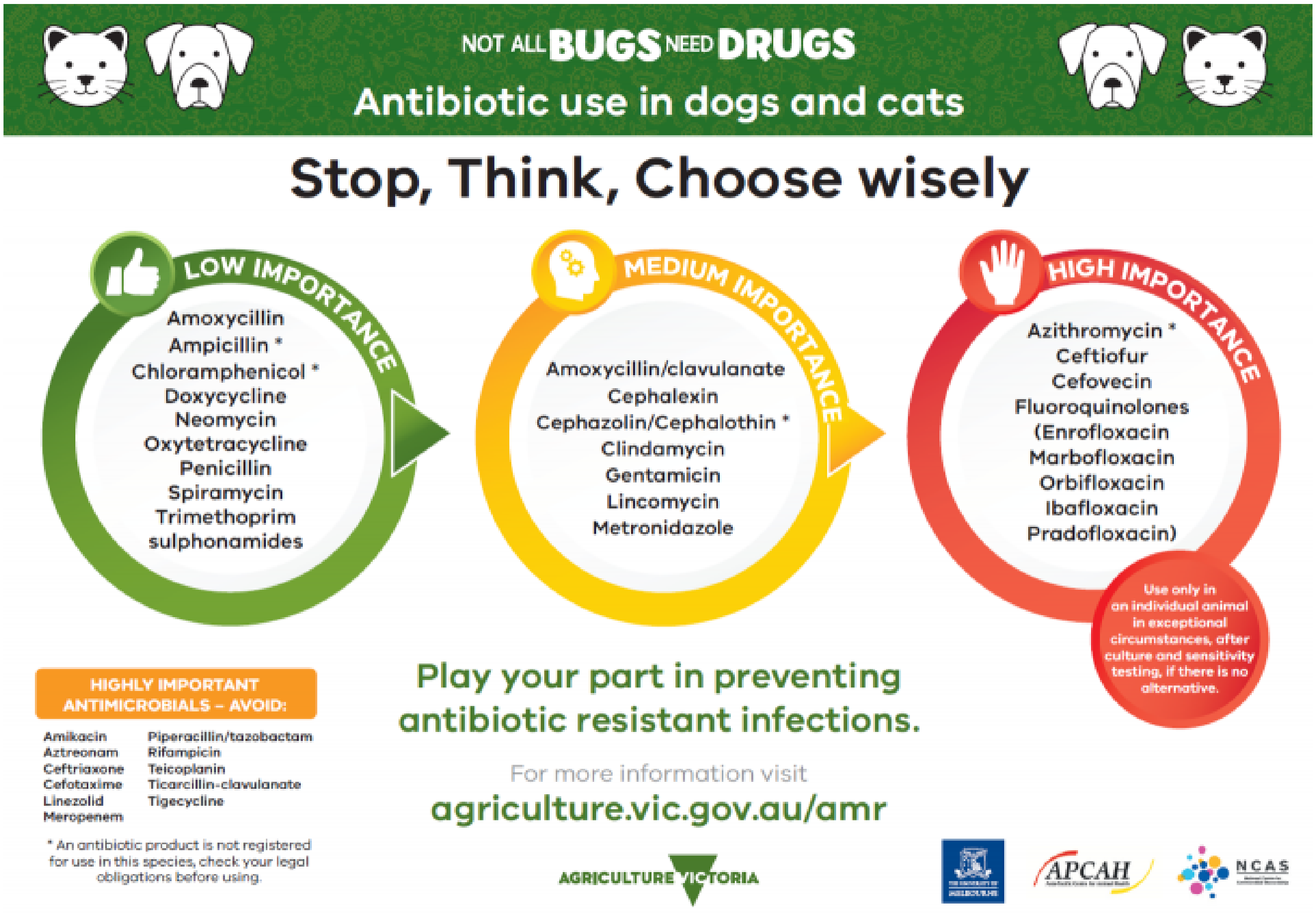

Have you heard of this rating system?

- Yes
- No

Do you use a traffic-light system for antimicrobial importance in your workplace?

- Yes
- No

In medical general practice, doctors have to get approval to prescribe high-importance antimicrobials to patients. This is not the case in veterinary practice. Do you agree that veterinarians should be able to prescribe antibiotics with **high importance to human medicine** without restrictions?

- Strongly agree
- Agree
- Somewhat agree
- Neither agree nor disagree
- Somewhat disagree
- Disagree
- Strongly disagree

Referring to the graphic above, if there were to be restrictions placed on veterinary prescribing of antimicrobials, which antimicrobials should they apply to?

	Strongly agree	Somewhat agree	Neither agree nor disagree	Somewhat disagree	Strongly disagree
All antimicrobials	○	○	○	○	○
Antimicrobials with medium and high importance rating	○	○	○	○	○
All antimicrobials with high importance rating	○	○	○	○	○
All high importance rating antimicrobials except 3rd generation cephalosporins (ceftiofur & cefovecin) and fluoroquinolone (enrofloxacin, marbofloxacin etc)	○	○	○	○	○

	Strongly agree	Somewhat agree	Neither agree nor disagree	Somewhat disagree	Strongly disagree
All high importance rating antimicrobials except 3rd generation cephalosporins (ceftiofur & cefovecin)	○	○	○	○	○
All high importance rating antimicrobials except fluoroquinolone (enrofloxacin, marbofloxacin etc)	○	○	○	○	○

If there were to be restrictions placed on veterinary prescribing of antimicrobials with **high importance** rating , which of the following do you think is appropriate?

	Strongly agree	Somewhat agree	Neither agree nor disagree	Somewhat disagree	Strongly disagree
These antimicrobials must not be allowed to be used in veterinary medicine under any circumstance	○	○	○	○	○
These antimicrobials must never allowed to be used in food-producing animals but can be used in other animals	○	○	○	○	○
Use can only proceed with approval from an independent office	○	○	○	○	○
Use in referral hospitals is allowed without approval	○	○	○	○	○
Use in general practice requires approval from an independent office	○	○	○	○	○
Use is only allowed after culture and susceptibility testing confirms that the pathogen is resistant to all low and medium rated antimicrobials that could be used to treat the case	○	○	○	○	○
Use is allowed after treatment failure with a lower importance rating antimicrobials	○	○	○	○	○
Use is allowed in critically ill animals	○	○	○	○	○
Other restrictions	○	○	○	○	○

What other restrictions do you think are appropriate?

Please indicate how strongly you agree with the following statements:

Approval is still required prior to high importance drugs being used in veterinary medicine:

	Agree	Unsure	Disagree
If culture and susceptibility testing confirms that the pathogen is resistant to all low and medium rated antimicrobials that could be used to treat the case	○	○	○
If treatment has failed with a lower importance rating antimicrobial	○	○	○
in critically ill animals	○	○	○

Would you like to tell us anything else about the role in veterinary medicine of antimicrobials with high importance to human medicine?

SOME CASE SCENARIOS

A cat presents with a draining abscess on its face. While the cat is febrile and inappetent and you believe that antimicrobial therapy is indicated, you do not think that the infection is life-threatening. Under which circumstances is it reasonable to treat this cat with cefovecin (long-acting 3rd generation cephalosporin), an antimicrobial with a high importance rating?

	Strongly agree	Somewhat agree	Neither agree nor disagree	Somewhat disagree	Strongly disagree
Never	○	○	○	○	○

	Strongly agree	Somewhat agree	Neither agree nor disagree	Somewhat disagree	Strongly disagree
If the cat is difficult to medicate	O	O	O	O	O
If you give the owner options of either oral or injectable antimicrobials and they choose cefovecin	O	O	O	O	O
If culture and susceptibility testing indicates that the pathogen is resistant to all low and medium rated antimicrobials that could be used to treat the case	O	O	O	O	O
With approval from an independent office	O	O	O	O	O
Always	O	O	O	O	O

A horse presents with a septic fetlock joint that could be life-threatening. Under which circumstances is it reasonable to inject the joint with amikacin, a high importance rated aminoglycoside?

	Strongly agree	Somewhat agree	Neither agree nor disagree	Somewhat disagree	Strongly disagree
Never	O	O	O	O	O
If culture and susceptibility testing indicates that the pathogen is resistant to gentamicin	O	O	O	O	O
If the owner can afford it	O	O	O	O	O
With approval from an independent office	O	O	O	O	O
Always	O	O	O	O	O

How would the following scenarios affect your decision about the above horse:

	Makes no difference	Makes some difference	Not sure	Should be taken into account	Any antimicrobial should be allowed
If the horse was the leading thoroughbred stallion in Australia	O	O	O	O	O
The prognosis was very poor	O	O	O	O	O
The horse is insured	O	O	O	O	O
The owners demand amikacin	O	O	O	O	O
Other treatments have failed	O	O	O	O	O

	Makes no difference	Makes some difference	Not sure	Should be taken into account	Any antimicrobial should be allowed
You practice in a region where horses are considered a food-producing species	○	○	○	○	○

A dairy cow presents with pneumonia and you believe antimicrobial therapy is indicated although the infection is not life-threatening. Under which circumstances is it reasonable to treat this cow with ceftiofur (3rd generation cephalosporin), an antimicrobial with a high importance rating?

	Strongly agree	Somewhat agree	Neither agree nor disagree	Somewhat disagree	Strongly disagree
Never	○	○	○	○	○
If culture and susceptibility testing indicates that the pathogen is resistant to all low and medium rated antimicrobials that could be used to treat the case	○	○	○	○	○
With approval from an independent office	○	○	○	○	○
Always, as ceftiofur is labelled to treat pneumonia in cattle	○	○	○	○	○
Always, as ceftiofur carries a nil milk with-holding period	○	○	○	○	○

Thanks for completing the survey. Do you have anything else you'd like to tell us?

Powered by Qualtrics
